# Iron and iron-related proteins in alcohol consumers: cellular and clinical aspects

**DOI:** 10.1007/s00109-022-02254-8

**Published:** 2022-10-10

**Authors:** Kevin Ferrao, Najma Ali, Kosha J. Mehta

**Affiliations:** 1 GKT School of Medical Education, Faculty of Life Sciences and Medicine, King’s College London, London, UK; 2 Centre for Education, Faculty of Life Sciences and Medicine, King’s College London, London, UK

**Keywords:** Iron, Alcohol, Alcohol-associated liver disease, ALD, Hepcidin, Ferritin, Transferrin, Carbohydrate-deficient transferrin, Transferrin receptor-1, Hemoglobin

## Abstract

Alcohol-associated liver disease (ALD) is one of the most common chronic liver diseases. Its pathological spectrum includes the overlapping stages of hepatic steatosis/steatohepatitis that can progress to liver fibrosis and cirrhosis; both are risk factors for hepatocellular carcinoma. Moreover, ALD diagnosis and management pose several challenges. The early pathological stages are reversible by alcohol abstinence, but these early stages are often asymptomatic, and currently, there is no specific laboratory biomarker or diagnostic test that can confirm ALD etiology. Alcohol consumers frequently show dysregulation of iron and iron-related proteins. Examination of iron-related parameters in this group may aid in early disease diagnosis and better prognosis and management. For this, a coherent overview of the status of iron and iron-related proteins in alcohol consumers is essential. Therefore, here, we collated and reviewed the alcohol-induced alterations in iron and iron-related proteins. Reported observations include unaltered, increased, or decreased levels of hemoglobin and serum iron, increments in intestinal iron absorption (facilitated via upregulations of duodenal divalent metal transporter-1 and ferroportin), serum ferritin and carbohydrate-deficient transferrin, decrements in serum hepcidin, decreased or unaltered levels of transferrin, increased or unaltered levels of transferrin saturation, and unaltered levels of soluble transferrin receptor. Laboratory values of iron and iron-related proteins in alcohol consumers are provided for reference. The causes and mechanisms underlying these alcohol-induced alterations in iron parameters and anemia in ALD are explained. Notably, alcohol consumption by hemochromatosis (iron overload) patients worsens disease severity due to the synergistic effects of excess iron and alcohol.

## Introduction

Alcohol-associated liver disease (ALD) is one of the most prevalent types of chronic liver diseases (CLDs) [1] that majorly contributes to the increasing rate of CLD-related mortality in the world. ALD is a cause of concern not only on its own but also because alcohol consumption acts as a cofactor in accelerating and exacerbating the pathologies of other CLDs like iron overload hemochromatosis and chronic viral hepatitis [2]. Chronic alcohol consumption is a risk factor for the development of hepatocellular carcinoma (HCC) [3]. There are several challenges in ALD diagnosis. For example, the early stages are often asymptomatic, and at present, there is no specific laboratory biomarker or diagnostic test that can confirm the etiology (and therefore diagnosis) of ALD. Also, there is a frequent denial of alcohol abuse and underreporting of alcohol intake in these patients [4]. Challenges around disease management and therapeutics are aplenty including difficulties in achieving complete abstinence, issues with disease reversal at advanced stages, and high costs of ALD management [5].

Excess iron can accelerate and exacerbate the pathologies of various CLDs, including ALD. It can promote hepatic fibrosis, carcinogenesis, and metastasis [6–8] and can also alter mesenchymal stem cell physiology that may influence liver functionality under pathological states [9].

Both acute and chronic alcohol exposure can disrupt iron homeostasis by reducing the synthesis of hepcidin [10], the liver-secreted iron-hormone that regulates systemic iron levels in the body [11]. As such, even mild to moderate amounts of alcohol consumption can increase iron stores [12]. In a recent study that investigated the effect of moderate alcohol consumption, consumption of > 88 g/week showed increased liver iron and consumption of > 56 g/week associated with higher brain iron, which, in turn, associated with poorer cognitive functions [13]. Moreover, both excess iron (through Fenton reaction) and alcohol metabolism produce increased levels of reactive oxygen species (ROS). Thus, these can independently cause oxidative damage and act synergistically to accelerate disease pathology [14]. Like chronic alcohol consumption, excess iron is also a risk factor for HCC. Hepatic iron overload poses an increased risk of cirrhosis, which causes predisposition to HCC [3]. Furthermore, alcohol consumption frequently hinders erythropoiesis resulting in anemia [15]. Thus, an iron-alcohol link is evident.

Therefore, it is crucial to understand the status and role of iron and related proteins in alcohol consumers. Alcohol-induced iron-related alterations and mechanisms may help decipher the complex disease pathophysiology; help identify adjunct biomarkers for ALD diagnosis, disease staging, and prognosis [16]; and/or develop supplementary therapeutic strategies.

Accordingly, this review discusses the alcohol-induced systemic changes in iron and iron-related proteins and explains the reasons and mechanisms underlying the alterations.

### Recommendations for alcohol consumption

Global recommendations for alcohol consumption differ between countries. For instance, in the USA, for men and women, the upper limits are 196 g/week and 98 g/week, respectively [17]. In the UK, the National Health Service (NHS) (as in August 2022) recommends that both men and women should not exceed 112 g/week (based on the calculation that 1 unit equals 8 g of pure alcohol), and this volume should be spread across 3 or more days. Notably, the risk for alcoholic cirrhosis increases due to chronic alcohol consumption of 20–50 g/day for women and 60–80 g/day for men [18].

### Overview of iron-related parameters in alcohol consumers

In this review, the phrase “alcohol consumers” includes the following: people consuming varying amounts of alcohol, i.e., mild, moderate, and heavy drinkers, different types of drinkers such as social and frequent drinkers, both acute and chronic alcohol consumption, and alcohol use disorder as well as ALD patients.

The aim of this review is to discuss the generic effect of alcohol on iron parameters regardless of the volume consumed, frequency of consumption, diagnosis of ALD, or its stage. This review does not aim to compare iron-parameters between those consuming different volumes or differentiate based on the regularity of alcohol consumption or ALD status.

Table 1 lists the alcohol-induced alterations in serum iron levels and core iron-related proteins. There was no defined alcohol threshold for inclusion of studies in Table 1. This table impartially states the effect of alcohol consumption on the levels of iron and iron-related proteins regardless of the amount or frequency of alcohol consumed or the presence or stage of ALD.

**Table 1:** Overview of iron-related parameters in alcohol consumers

**Iron-related proteins and parameters in serum**	**Reference range or levels in healthy controls or as otherwise indicated** **Values shown as mean ± SEM or mean (SD) or median (range); *n* represents the number of samples/patients**	**Alcohol-induced systemic alterations** **Values shown as mean ± SEM or mean ± SD or mean (SD) or median (range); *n* represents the number of samples/patients**
**Iron**		**May remain within reference range or similar to corresponding controls**
(Essential for many cellular and physiological processes)	• Range in controls for inclusion: 11–26 µmol/L; Range detected in controls:17.55 ± 0.67 µmol/L; *n* = 30 [22] • Reference range: 10.7–26.9 µmol/L [21] • Range in controls: 87.7 ± 39.2 µg/dL (15.7 ± 7.0 µmol/L) (*n* = 20; alcohol consumption less than 20 g/day in women and 40 g/day in men) [25] • In controls: 16.2 (5.35–25.2) µmol/L (*n* = 30; social drinkers consumed less than 30 g/day (210 g/week)) [20]	• 18.03 ± 1.94 µmol/L (*n* = 24; ALD patients) [22] • 22.4 (12.8) µmol/L (*n* = 43; alcoholic cirrhosis); 21.7 (10.4) µmol/L (*n* = 302; alcoholic hepatitis) [21] • 121.4 ± 69.3 µg/dL (21.7 ± 12.4 µmol/L) (*n* = 25; ALD active drinkers without cirrhosis, average daily alcohol intake of 154 g) [25] • 19.6 (3.39–49.4) µmol/L (*n* = 148; alcohol-dependent patients, mean alcohol consumption of 1344 g/week) [20]
		**May be lower than corresponding controls**
	• Total iron in controls: 92.25 ± 33.93 µg/dL (16.51 ± 6.07 µmol/L) (*n* = 32; in non-alcoholics) [53]	• Total iron: 72.68 ± 43.61 µg/dL (13.01 ± 7.81 µmol/L) (*n* = 238; heavy alcohol consumers drinking 199 ± 115 g/day during the last 31 ± 12 years) [53]
		**May be higher than corresponding controls or at upper end of reference range**
	• Reference range: 59–158 µg/dL (10.6–28.3 µmol/L) [46] • In non-drinkers (men): 19.9 ± 6.1 µmol/L (*n* = 267; drank 2–3 times/year or less, or did not drink at all) [80] • Hemochromatosis patients that consumed < 60 g/day alcohol: 36 (7.4) µmol/L; *n* = 345 [79]	• 157 µg/dL (28.1 µmol/L) (*n* = 235; ALD patients) [46] • 24.4 ± 8.6 µmol/L (men) (*n* = 68; drank twice/week or more often) [80] • Hemochromatosis patients that consumed ≥ 60 g/day alcohol: 39.9 (6.3) µmol/L; *n* = 33 [79]
**Ferritin**		**May remain within reference range or similar to corresponding controls**
(Iron-storing protein; typically reflect iron stores; present in circulation and intracellularly) [63]	• Range in controls for inclusion: 30–250 µg/L; Detected levels in controls: 151.0 ± 13.83 µg/L; *n* = 30 [22]	• 296.22 ± 96.11 µg/L (*n* = 24; ALD patients) (Apparently high value in ALD patients but not statistically significant from controls) [22]
		**May be higher than corresponding controls or reference range**
	• Range in controls: 115.7 ± 81.8 µg/L (*n* = 20; alcohol consumption less than 20 g/day in women and 40 g/day in men) [25] • Under alcohol abstinence: 388 ± 237 µg/L (*n* = 13, after abstaining for 1.5 to 6 weeks) [77] • Reference range: 30–400 µg/L [46] • Range in controls: 258.74 ± 37.39 µg/L (*n* = 32; non-alcoholics) [53] • In controls: 83 (21.6–207.5) µg/L (*n* = 30; social drinkers consumed less than 30 g/day (210 g/week)) [20] • In non-dependents on alcohol (social drinkers): 72 µg/L (*n* = 115; alcohol non-dependents with a negative AUDIT score of ≤ 6/8 and negative diagnosis of alcohol dependence, as defined by ICD-10 and DSM-IV) [68] • Hemochromatosis patients that consumed < 60 g/day alcohol: 968.7 (1129.3) µg/L; *n* = 345 [79]	• 618.1 ± 436 µg/L (*n* = 25, ALD active drinkers without cirrhosis, average daily alcohol intake of 154 g) [25] • 1483 ± 1134 µg/L (58% of alcoholics showed increased ferritin); (*n* = 13, alcoholics before abstinence) 351 ± 493 µg/L (*n* = 75, alcoholic cirrhotic patients) 723 ± 803 µg/L (*n* = 36, alcoholic non-cirrhotic patients) [77] • 567.2 µg/L (*n* = 235; ALD patients) [46] • 543.23 ± 757.61 µg/L (*n* = 238; heavy alcohol consumers drinking 199 ± 115 g alcohol/day for past 31 ± 12 years) [53] • 277.6 (88.4–1592) µg/L; (*n* = 148; alcohol-dependent patients, mean alcohol consumption of 1,344 g/week) [20] • 247 µg/L (*n* = 101; alcohol dependent patients with a positive AUDIT score of > 6/8 and positive diagnosis for alcohol dependence, as defined by ICD-10 and DSM-IV) [68] • Hemochromatosis patients that consumed ≥ 60 g/day alcohol: 1745.2 (1792.1) µg/L; *n* = 33 [79] • A male patient with ALD and ferroportin disease showed serum ferritin level of 6574 µg/L [67] • 327 ± 440.6 µg/L in alcoholic cirrhosis patients with hepcidin < 8 µg/L and 567 ± 629.6 µg/L in alcoholic cirrhosis patients with hepcidin > 8 µg/L; *n* = 120 [32]
**Hepcidin**		**May be lower than corresponding controls or reference range**
(A liver-synthesized hormone in circulation that maintains systemic iron homeostasis) [28]	• In control subjects: 36.93 ng/mL; *n* = 8 [22] • Reference range: 17–286 ng/mL; *n* = 114 [84] • Pro-hepcidin levels in healthy controls: 1570 ± 260 ng/mL; *n* = 9 [31]	• 23.16 ng/ml (*n* = 16; ALD patients) 20.10 ng/mL (*n* = 2; ALD patients with iron overload) 22.67 ng/ml (*n* = 6; ALD patients without iron overload) 24.30 ng/mL (*n* = 8; ALD patients with anemia) [22] • Median = 8 µg/L (*n* = 237; alcoholic cirrhosis) [32] • Pro-hepcidin levels: 710 ± 540 ng/mL (*n* = 47, ALD patients) [31]
**Transferrin**		**May remain within reference range or similar to corresponding controls**
(Iron transporter in circulation) [66]	• In controls: 2.44 (1.79–3.54) g/L (*n* = 30; social drinkers consumed less than 30 g/day (210 g/week)) [20] • Reference range: 2–3.6 g/L [46]	• 2.33 (0.53–4.08) g/L (*n* = 148; alcohol-dependent patients, mean alcohol consumption of 1344 g/week) [20] • 2.5 g/L (*n* = 235, ALD patients) [46]
		**May be lower than corresponding controls or reference range, or at lower end of reference range**
	• Range in controls: 2.63 ± 1.29 g/L (*n* = 32; non-alcoholics) [53] • In controls: 3.1 ± 0.2 g/L (*n* = 6; healthy volunteers) [52]	• 1.9 ± 0.64 g/L (*n* = 238; heavy alcohol consumers drinking 199 ± 115 g/day during the last 31 ± 12 years) [53] • 1.8 ± 0.3 g/L in alcoholic cirrhosis (*n* = 5); 2.8 ± 0.2 g/L in alcoholic fatty liver (*n* = 6) [52]
**Transferrin saturation (TSAT)**		**May remain similar to corresponding controls**
(Reflects saturation of transferrin with iron; A diagnostic index for iron deficiency or iron overload) [85]	• In controls: 31.22 ± 0.96%; *n* = 30 [22] • Transferrin saturation index: 29.45 ± 17.35%; *n* = 32; non-alcoholics [53] • Healthy controls: Approximate range: 20–45%; *n* = 60 [23]	• 32.22 ± 4.88% (*n* = 24; ALD patients) [22] • Transferrin saturation index: 31.90 ± 24.98% (*n* = 238; heavy alcohol consumers drinking 199 ± 115 g/day during the last 31 ± 12 years) [53] • Approximate range: 20–45% (*n* = 13; alcoholic fatty liver patients with an intake of 40 g/day) [23]
		**May be higher than corresponding controls or reference range**
	• Range in controls: 27.8 ± 12.8% (*n* = 20; alcohol consumption less than 20 g/day in women and 40 g/day in men) [25] • 30.0 ± 10.4% in non-drinkers (men) (*n* = 267; drank 2–3 times/year or less, or did not drink at all) [80] • In controls: 27.32% (9.11–50.22) (*n* = 30; social drinkers consumed less than 30 g/day (210 g/week)) [20] •Hemochromatosis patients that consumed < 60 g/day alcohol: 80.1% (13.7); *n* = 345 [79]	• 39.7 ± 20.9% (*n* = 25; ALD active drinkers without cirrhosis, average daily alcohol intake of 154 g) [25] • 35.8 ± 13.1% (men) (*n* = 68; drank twice/week or more often) [80] • 33.22% (6.02–122.3) (*n* = 148, alcohol-dependent patients, mean alcohol consumption of 1,344 g/week) [20] • Hemochromatosis patients that consumed ≥ 60 g/day alcohol: 87.1% (9.3); *n* = 33 [79] • 36 ± 24% in alcoholic cirrhosis (*n* = 75); 38 ± 22% in non-cirrhotic alcoholics (*n* = 36) [77] • 43.1 ± 24.8% in alcoholic cirrhosis patients with hepcidin > 8 µg/L (*n* = 120) and 50.8 ± 46.9% in alcoholic cirrhosis patients with hepcidin < 8 µg/L (*n* = 117) [32]. Here, there was no significant difference between the two groups, but the range of values in both groups appeared to be higher than the reference ranges in controls/non-drinkers stated in other studies
**Carbohydrate-deficient transferrin**		**May remain similar to corresponding controls**
(Biomarker of chronic alcohol consumption) [69, 70]	• In non-dependents on alcohol: 2.27% (0.36); *n* = 6 [86]	• 2.33% (0.61) (*n* = 21; alcohol dependents) [86]
		**May be higher than corresponding controls**
	• In controls: 1.95% (1.25–3.31) (*n* = 30; social drinkers consumed less than 30 g/day (210 g/week)) [20] • In non-dependents on alcohol (social drinkers): 2.2% (*n* = 115; alcohol non-dependents with a negative AUDIT score of ≤ 6/8 and negative diagnosis of alcohol dependence, as defined by ICD-10 and DSM-IV) [68] • Average disialotransferrin to total transferrin fraction: 1.2% (0.20) [87] • In non-drinkers: 1.71 ± 0.04%; *n* = 29 [88]	• 6.27% (1.26–17.66) (*n* = 148; alcohol-dependent patients, mean alcohol consumption of 1344 g/week) [20] • 3.9% (*n* = 101; alcohol dependent patients with a positive AUDIT score of > 6/8 and positive diagnosis for alcohol dependence, as defined by ICD-10 and DSM-IV) [68] • Upper limit of reference interval for disialotransferrin to total transferrin fraction (in non-drinkers and light and heavy drinkers combined): 1.70% [87] • Heavy drinkers: 2.44 ± 0.14% (*n* = 29; consuming ≥ 420 g alcohol/week) [88]
**Soluble transferrin receptor (sTfR)**		**May remain similar to corresponding controls**
(Unknown function; elevated in the serum in iron deficiency anemia) [20]	• Reference range: 2.2–5.0 mg/L; In controls: 2.65 (1.9–3.7) mg/L (*n* = 30; social drinkers consumed less than 30 g/day (210 g/week)) [20] • In non-dependents on alcohol (social drinkers): 2.9 mg/L (*n* = 115; alcohol non-dependents with a negative AUDIT score of ≤ 6/8 and negative diagnosis of alcohol dependence, as defined by ICD-10 and DSM-IV) [68]	• 2.70 (1.2–7.1) mg/L (*n* = 148; alcohol-dependent patients, mean alcohol consumption of 1344 g/week) [20] • 3.2 mg/L (*n* = 101; alcohol dependent patients with a positive AUDIT score of > 6/8 and positive diagnosis for alcohol dependence, as defined by ICD-10 and DSM-IV) [68]
**Hemoglobin**		**May remain within reference range or similar to corresponding controls**
(Oxygen transport protein)	• Reference range: 11.17–17.0 g/dL [70] • Range in controls: 1.4 ± 0.1 g/dL (*n* = 20; alcohol consumption less than 20 g/day in women and 40 g/day in men) [25] • In non-dependents on alcohol (social drinkers): 14.2 g/dL (*n* = 115; alcohol non-dependents with a negative AUDIT score of ≤ 6/8 and negative diagnosis of alcohol dependence, as defined by ICD-10 and DSM-IV) [68]	• 12–18 g/dL in 82.5% of patients; 13.35 g/dL (2.16) (total number of patients = 103); consumption of at least 16 g/day or 2 units (drinks)/day) [47] • 1.4 ± 0.2 g/dL (*n* = 25; ALD active drinkers without cirrhosis, average daily alcohol intake of 154 g) [25] • 14.6 g/dL (*n* = 101; alcohol dependent patients with a positive AUDIT score of > 6/8 and positive diagnosis for alcohol dependence, as defined by ICD-10 and DSM-IV) [68]
		**May be lower than corresponding controls or at lower end of reference range**
	• Reference range: 11.5–18 g/dL; 14.0 ± 0.31 g/dL in controls; *n* = 30 [22] •13.9 ± 1.53 g% (*n* = 77; healthy controls) [45]	• 12.66 ± 0.44 g/dL (*n* = 24; ALD patients) [22] • 12.0 ± 2.87 g% in ALD (*n* = 40; consumed ≥ 80 g/day for at least 5 years) [45] • Less than 12 g/dL in 17.5% of patients (total number of patients = 103); consumption of at least 16 g/day or 2 units (drinks)/day) [47]
		**May be higher than corresponding controls**
	• In controls: 13.5 g/dL (12.5–16.1) (*n* = 30; social drinkers consumed less than 30 g/day (210 g/week)) [20] • 13.2 ± 1.8 g/dL (*n* = 76; biliary acute pancreatitis) [89] • In men who consumed less than 14 drinks/week: 9.48 ± 0.62 mmol/L (15.3 ± 0.9 g/dL); *n* = 364 [90] • In women who consumed less than 7 drinks/week: ranging from 8.33 ± 0.57 mmol/L to 8.62 ± 0.59 mmol/L (13.42 ± 0.92 g/dL to 13.89 ± 0.95 g/dL) [90]	• 14.7 g/dL (9.0–17.5) (*n* = 148; alcohol-dependent patients, mean alcohol consumption of 1344 g/week) [20] • 14.7 ± 3.1 g/dL (*n* = 50; alcohol-induced acute pancreatitis) [89] • In men, who consumed more than 14 drinks/week: 9.60 ± 0.63 mmol/L (15.5 ± 1.0 g/dL); *n* = 146 [90] • In women who consumed more than 7 drinks/week: 8.48 ± 0.41 mmol/L to 8.83 ± 0.55 mmol/L (13.66 ± 0.66 g/dL to 14.23 ± 0.89 g/dL) [90]

## What is the status of serum iron in alcohol consumers/ALD patients? Normal, low, or high

A vast majority of chronic heavy alcohol consumers develop alcoholic fatty liver, which is an early stage of the ALD disease spectrum. However, only a minority (10–20%) from this group develop advanced ALD, alluding to the idea that other factors such as iron levels may contribute to the ALD pathogenesis [19]. Essentially, these patients show a broad range of effect on serum iron, i.e., they can show either unaltered serum iron levels, iron deficiency, or serum iron-loading (Table 1).

Some ALD patients have shown serum iron levels similar to controls [20–22]. Moreover, a study showed similar levels of serum iron in patients with ALD cirrhosis, alcoholic hepatitis, and healthy volunteers that did not demonstrate any acute or chronic health conditions and consumed alcohol within the recommended guidelines [21]. This implied that serum iron levels were similar between (a) ALD patients and healthy controls who consumed alcohol within the recommended guidelines and (b) between certain stages of the ALD pathological spectrum. Distinct from this, some ALD patients show iron deficiency [12] or anemia (low hemoglobin or red blood cells) [22]. The plausible causes of iron deficiency and anemia in ALD have been discussed in the subsequent section.

Contrasting these examples, chronic alcohol consumption can elevate serum iron levels, and this can be found in about 64% of ALD cases, according to a report [23]. Consumption of more than 2 alcoholic drinks per day can increase the risk of iron overload [12]. Relatedly, ALD patients/chronic alcohol consumers can show increments in intestinal iron absorption (by twofold), serum ferritin, iron saturation of transferrin, and hepatic iron stores [12, 22, 24].

## What causes alcohol-induced serum iron elevation?

### Hepcidin decrement and its desensitization to iron elevation

Alcohol-induced downregulation of hepcidin synthesis in the liver leading to low circulatory hepcidin is the main cause of elevated serum iron in ALD (Table 1) [10, 22, 25]. Reduced serum hepcidin is observed in about 60% of ALD cases [23].

Hepcidin is the master regulator of systematic iron homeostasis. It is synthesized predominantly by the liver hepatocytes and secreted into the circulation in response to elevation in systemic iron levels and/or inflammation. Hepcidin functions by binding to the sole known mammalian cellular iron-exporter ferroportin. Ferroportin is present on the cell surfaces of various cell types including the duodenal enterocytes, hepatocytes, and macrophages, cells that play a crucial role in iron regulation. Binding of hepcidin to ferroportin causes internalization and degradation of both hepcidin and ferroportin. This prevents iron egress into the circulation. Thus, when systemic iron loading occurs, hepcidin secretion by the liver is increased. Hepcidin prevents iron entry into the circulation by inhibiting intestinal iron absorption and by preventing the release of iron from iron-storing/iron recycling cells in the body [26–30].

Both chronic and acute alcohol exposure can reduce liver hepcidin expression in the hepatocytes, thereby reducing circulatory hepcidin (Table 1) [10, 22, 24]. Also, ALD patients showed reduced levels of serum prohepcidin (precursor of hepcidin) [31]. Resultantly, there is uncontrolled iron entry into the circulation from the enterocytes [27] and the iron-storing and recycling cells, collectively leading to elevation in serum iron levels, as observed in some ALD patients.

Under physiological conditions, elevation in serum or liver iron upregulates hepcidin expression, i.e., hepcidin level, and its regulation is sensitive to iron levels [28]. Reduced hepcidin levels were seen not only in ALD cases with anemia but also in ALD cases with and without iron overload. Here, the ALD cases showed serum iron levels similar to controls [22]. This suggests that alcohol exposure desensitizes hepcidin transcription to iron levels and suppresses it [24].

Notably, hepcidin can be downregulated in hepatocellular carcinoma. Alcoholic cirrhosis patients with low levels of hepcidin show a high risk of hepatocellular carcinoma and death. In this group, low levels of hepcidin associate with poor long-term survival [29, 32].

### Elevation of duodenal iron transporters

Another mechanism that supports systemic iron acquisition involves duodenal divalent metal ion transporter-1 (DMT1). DMT1 is the iron transport protein on the apical region of the duodenal enterocytes. It transports elemental iron from the gut lumen into the enterocytes.

In animal models, alcohol exposure downregulated hepcidin expression in the liver, which led to elevated expression of DMT1 and ferroportin in the duodenum [33]. Also, increased DMT1 and ferroportin mRNA expressions were observed in duodenal biopsies of ALD patients; more pronounced in those without iron-overload in comparison with those with iron-loading and in ALD anemic patients compared to controls [22]. Thus, increased DMT1 expression on the apical region along with increased ferroportin on the basolateral surface of the duodenal enterocyte can facilitate further iron entry into the circulation of ALD patients.

The tendency of higher level of iron acquisition by anemic ALD patients (i.e., those showing reduced red blood cell count or hemoglobin) may be a rescue attempt aiming to make iron available for the developing erythrocytes for hemoglobin synthesis. Distinct from this, higher level of iron acquisition by those without iron-overload probably reflects or is the result of the alcohol-induced dysregulation of iron homeostasis.

## What are the cellular mechanisms underlying alcohol-induced hepcidin downregulation?

### The mechanisms

**Fig. 1:**
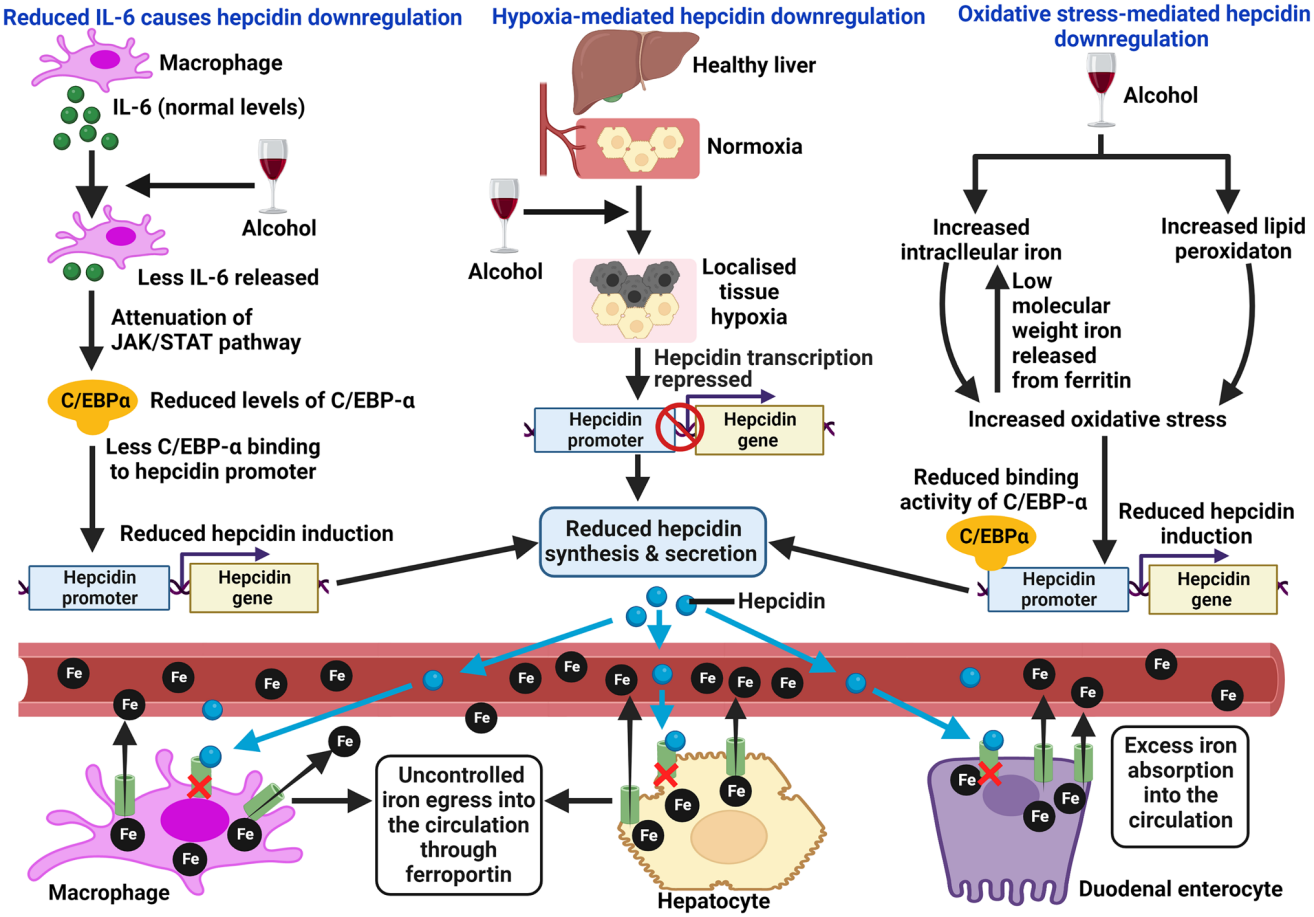
Mechanisms of alcohol-induced hepcidin downregulation. The figure depicts a few mechanisms of alcohol-induced hepcidin suppression. These include events induced by hypoxia, oxidative stress, and reduced IL-6. Indeed, there are other hepcidin-suppressing mechanisms that may work alongside these events. Hepcidin downregulation leads to uncontrolled iron entry into plasma via ferroportin. Red crosses inside the macrophage, hepatocyte, and duodenal enterocyte indicate normal mechanism of hepcidin-mediated degradation of ferroportin (shown as green structures), which prevents iron entry into the circulation and thereby regulates systemic iron homeostasis under excess iron conditions. The normal hepcidin-ferroportin axis is disturbed due to alcohol-induced hepcidin suppression. C/EBP-α: CCAAT/enhancer-binding protein alpha. The figure was created with BioRender.com

Firstly, alcohol-induced oxidative stress suppresses hepcidin expression [33]. Basically, alcohol metabolism generates ROS and lipid peroxidation products. Simultaneously, excess iron accelerates the Fenton reaction and generates large amounts of ROS. Also, oxidative stress can release low-molecular weight iron from ferritin [34]. Thus, both alcohol and iron can independently cause oxidative stress and the two together can amplify the deleterious effects in chronic alcohol consumers. Oxidative stress can inhibit hepcidin promoter activity, i.e., reduce the DNA-binding activity of the transcription factor C/EBP-α [10], which is known to regulate (stimulate) hepcidin expression [35]. Thus, hepcidin transcription in the hepatocytes is reduced (Fig. 1). Moderate alcohol consumption modulates hepatic hepcidin expression and thereby systemic iron homeostasis through oxidative stress without causing evident liver injury [24].

Another mechanism involves reduction in the expression of C/EBP-α. IL-6 is one of the inducers of hepcidin expression via the JAK/STAT pathway, and suppression of IL-6 attenuates hepcidin expression. Ethanol suppresses the release of IL-6 from macrophages [36]. Reduced IL-6 attenuates JAK/STAT signaling, decreases C/EBP-α expression and its binding to DNA, thereby attenuating hepcidin induction and reducing hepcidin concentration in the circulation [24, 34] (Fig. 1).

In addition, alcohol metabolism in the liver induces localized hypoxia [37], which is known to repress hepatic hepcidin induction [38]. Also, acetaldehyde, a by-product of ethanol metabolism, has shown to reduce hepcidin expression in vitro [33]. Moreover, in alcohol-fed mice, the activity of the BMP-SMAD pathway (main pathway for hepcidin induction in response to iron) was repressed. There was attenuation of binding of SMAD4 to hepcidin promoter, which hindered hepcidin transcription [39].

### Inflammation in ALD, but still no hepcidin upregulation?

Iron-induced hepcidin synthesis occurs via the BMP-SMAD-1/5/8 pathway. However, inflammation-induced hepcidin synthesis occurs via IL6-JAK-STAT3 pathway as well as the BMP-SMAD pathway. Studies suggest that during inflammation, the JAK-STAT3 and BMP-SMAD pathways interact with each other to induce hepcidin [11].

Chronic inflammation is often present in ALD [19]. In theory, this should lead to increased hepcidin synthesis and secretion because hepcidin is induced by inflammation. However, this is not the case in ALD despite the presence of inflammation (Table 1). One reason for this could involve the BMP-response element. A BMP-response element and the STAT3 response element on the hepcidin promoter are in close vicinity of each other. Previously, it was observed that inactivation of the BMP-response element repressed IL6-mediated induction of hepcidin expression. Based on this, it has been proposed that alcohol-induced inactivation of the BMP-response element may suppress the inflammation-induced upregulation of hepcidin in ALD [40].

There may be additional mechanisms that prevent inflammation-induced hepcidin upregulation under the influence of alcohol. It is highly possible that such mechanisms work together with the aforementioned cellular mechanisms of alcohol-induced hepcidin downregulation to produce the final effect of hepcidin decrement.

## Plausible causes of iron deficiency in alcohol consumers

While alcohol consumption can promote systemic iron loading, a proportion of alcohol consumers/ALD patients have been reported to be iron deficient. Malnutrition is frequently observed among alcoholics, which may partly explain iron deficiency in some subjects. However, there could be other reasons for this. Chronic and excessive alcohol consumption not only overwhelms the liver (due to alcohol metabolism here) but also damages the gastrointestinal tract by causing intestinal inflammation, duodenal erosions, breakage of the integrity of the intestinal mucosa, and duodenal hemorrhage [41–43]. While these events may promote the translocation of bacterial endotoxins into the circulation (portal blood), these may also hamper the iron absorption process in the duodenum, and thereby cause iron deficiency. Based on this, it can be assumed that the status of iron in alcohol consumers-iron deficiency or iron overload may partly depend on the intestinal health of the subjects, which can vary between subjects due to its dependence on various factors. This is a hypothesis and needs to be tested. If correct, then the mechanisms underlying iron-deficiency in alcoholics need to be deciphered.

## Prevalence of anemia in ALD patients

The World Health Organization defines anemia as a condition wherein the number of red blood cells (RBCs) or hemoglobin concentration is lower than normal. Some ALD patients/alcohol consumers have shown iron deficiency (iron levels lower than normal) and/or iron deficiency anemia (anemia caused due to low levels of iron) [12, 44].

Alcohol can elevate or reduce hemoglobin levels or levels may remain unaltered (Table 1). Thus, some ALD patients can show low levels of hemoglobin, RBC count, and lower than normal hematocrit [45, 46]. In a study, hemoglobin levels were < 12 g/dL in 17.5% of patients with alcohol use disorder (subjects consumed a minimum of 16 g of alcohol/day in this study) [47]. Interestingly, in some ALD cases, serum iron parameters were low without overt iron deficiency anemia, where the criteria for iron deficiency anemia was “(serum ferritin < 20 μg/L, hemoglobin < 11 g/dL, and transferrin saturation < 16%)” [22]. Prevalence of iron deficiency in both alcoholic hepatitis and cirrhotic ALD patients has been similar [21], reiterating that iron status may not drastically differ between the different stages of the ALD pathological spectrum.

An investigation showed that anemia existed in about 41% of patients with alcohol-related etiology and suggested that 81% of patients with chronic ALD suffer from anemia [48]. This is not surprising because anemia is seen from about two-thirds up to about 75% patients in the advanced stages of chronic liver disease [44, 48].

## What causes anemia in ALD?

**Fig. 2:**
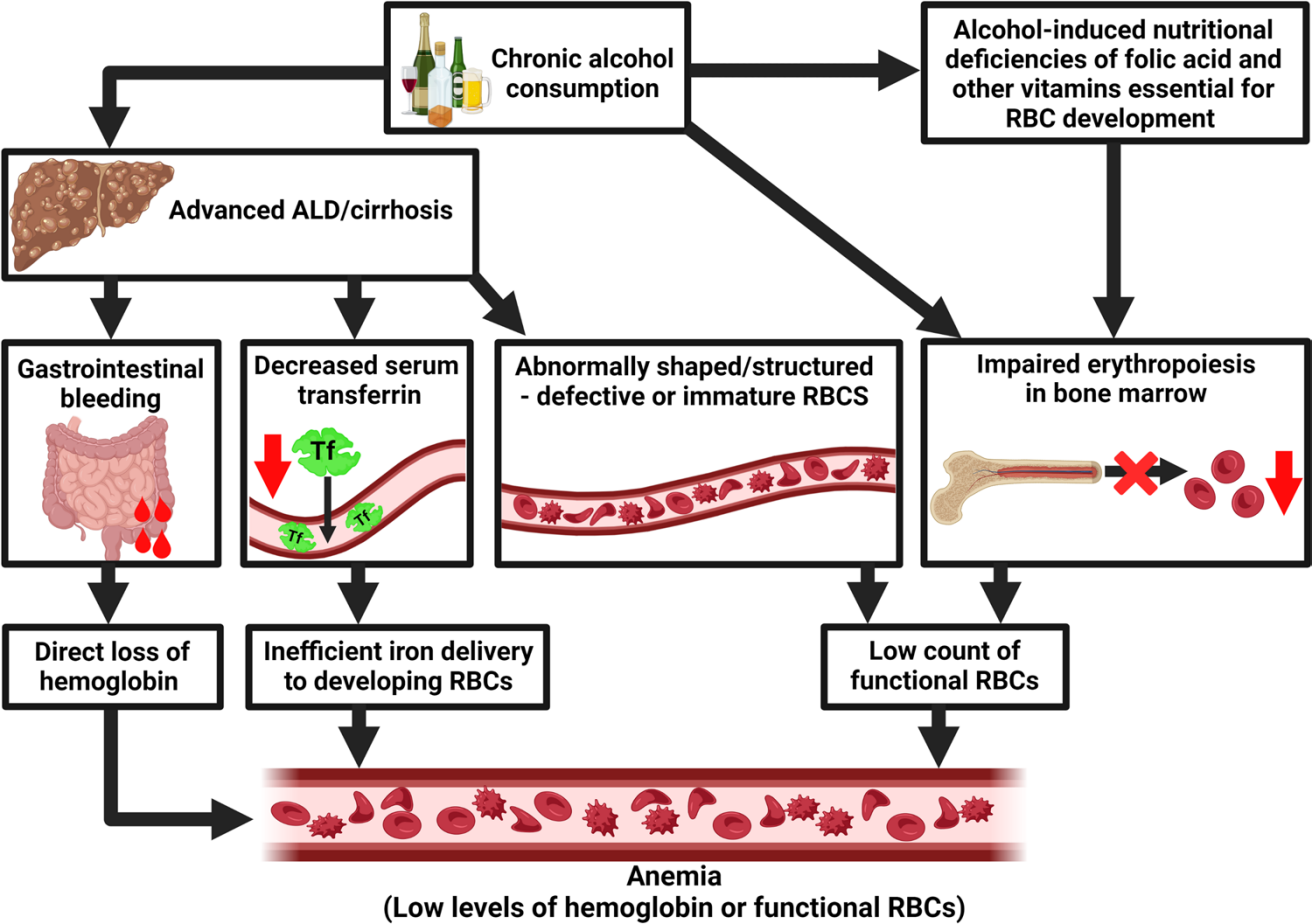
Events causing alcohol-induced anemia. There are several alcohol-induced events that can lead to anemia, for example, alcohol-induced nutritional deficiencies (e.g., B12 and B9 deficiencies) and impaired erythropoiesis in the bone marrow. Also, alcohol directly exerts toxic effects and reduces the number of RBC precursors in the bone marrow. Chronic alcohol consumption can produce defective RBCs that are swiftly destroyed via hemolysis. Moreover, loss of liver functionality can decrease serum transferrin levels leading to inefficient iron delivery to developing RBCs. Gastrointestinal bleeding in advanced ALD leads to direct loss of RBCs resulting in anemia. The figure was created with BioRender.com

The varied reasons for anemia in alcohol consumers have been summarized in Fig. 2. It is important to note that in ALD, many of the anemia-causing mechanisms may co-exist and ALD patients may show anemia with iron deficiency. Hospitalized chronic alcoholic consumers commonly show iron deficiency, increased mean corpuscular volume, and megaloblastic and sideroblastic anemias. However, in cases of chronic alcohol consumption, adequate diet can be protective, implying that alcohol per se does not cause iron deficiency or anemia [10].

### The erythrocyte context

Alcohol consumption increases the risk of gastrointestinal bleeding in alcoholic cirrhosis patients with portal hypertension. So, blood loss via upper gastrointestinal tract bleeding can cause anemia and iron deficiency [49]. Secondly, excessive alcohol intake has a direct toxic effect on the bone marrow. It suppresses hematopoiesis (reversibly), which can lead to anemia [44]. RBC count in alcoholics was found to be significantly reduced, although within the normal range [20]. Thirdly, alcohol can affect hematopoiesis either by directly damaging erythroid precursors leading to enlarged erythrocytes (macrocytosis) or by interfering with heme synthesis. Essentially, ethanol interferes with the activity of an enzyme that is critical for hemoglobin synthesis, which causes iron to accumulate as ferritin inside RBC precursors. These precursors are unable to mature into functional RBCs, thereby leading to sideroblastic anemia, as observed in about 33% of severe alcoholics. Abstinence from alcohol usually reverses this and restores RBC levels to normal [15, 45]. Moreover, there could be a nutrition-related reason for anemia. Alcohol consumption causes nutritional deficiencies such as those related to folic acid and other B vitamins due to poor intestinal absorption. This can adversely affect RBC development and cause anemia [44].

Heavy and chronic alcohol consumption can lead to several types of hemolytic anemias, for example, spur cell anemia. Spur cell anemia shows the presence of increased numbers of large RBCs with spike-like projections. It has been observed in alcohol-induced liver cirrhosis [50], and it occurs in about 3% of alcoholics with advanced liver disease [15]. The distortion in RBC shape is caused by excess amounts of cholesterol incorporated into the cell membrane of RBCs, which greatly increases the surface area of these cells without corresponding increase in cell volume. These cells are prematurely eliminated by splenic macrophages, leading to a low count of normal/functional RBCs [15]. Spur cell anemia in ALD patients needs to be carefully managed to prevent organ failure, and the patients are required to abstain from alcohol to prevent progression to liver cirrhosis [50]. ALD patients may also develop stomatocyte hemolysis, wherein RBCs with defective cell membranes develop stoma-like shape (RBCs called as stomatocytes), and these are trapped and destroyed in the spleen. In about 25% of alcoholics, the number of stomatocytes greatly increases but the condition can be reversed by alcohol abstinence. Rarely, alcoholics show hypophosphatemia-induced hemolysis, which is caused by low phosphate levels in the blood. Basically, alcohol causes phosphate excretion via urine, and when this occurs in excess, levels of phosphate and ATP in the RBCs substantially decrease leading to increased rigidity of RBC membranes. These damaged RBCs are destroyed in the spleen, causing hemolytic anemia [15].

Essentially, via various mechanisms, chronic excessive alcohol consumption directly reduces the number of blood cell precursors in the bone marrow or causes structural abnormalities in blood cell precursors resulting in lesser than normal number of functional/mature RBCs (and also other blood cells) [15, 44], and this causes anemia.

### The transferrin context

Yet another reason for alcohol-induced anemia could be related to the levels of circulating transferrin. Transferrin delivers iron to various cells of the body by binding to membrane-bound transferrin receptor-1 (TfR1). Unlike iron deficiency anemia wherein transferrin levels are increased, anemia of chronic diseases and anemia of mixed origin tend to show normal/low and low transferrin levels, respectively [51].

While serum transferrin level remained unaltered or within the reference range in some chronic alcohol consumers and ALD patients [20, 46], it was found to be decreased (compared to controls) in severe alcoholics/ALD patients, specifically in patients with alcoholic cirrhosis (Table 1) [52, 53] and severe alcoholic hepatitis [54]. Reduced serum transferrin was recognized as an independent predictor of mortality [54]. Low transferrin levels in presence of normal or low serum iron levels [53] may contribute to iron deficiency anemia in alcoholics because insufficient transferrin level impairs iron delivery to the developing erythrocytes, and thereby restricts hemoglobin synthesis.

**Fig. 3:**
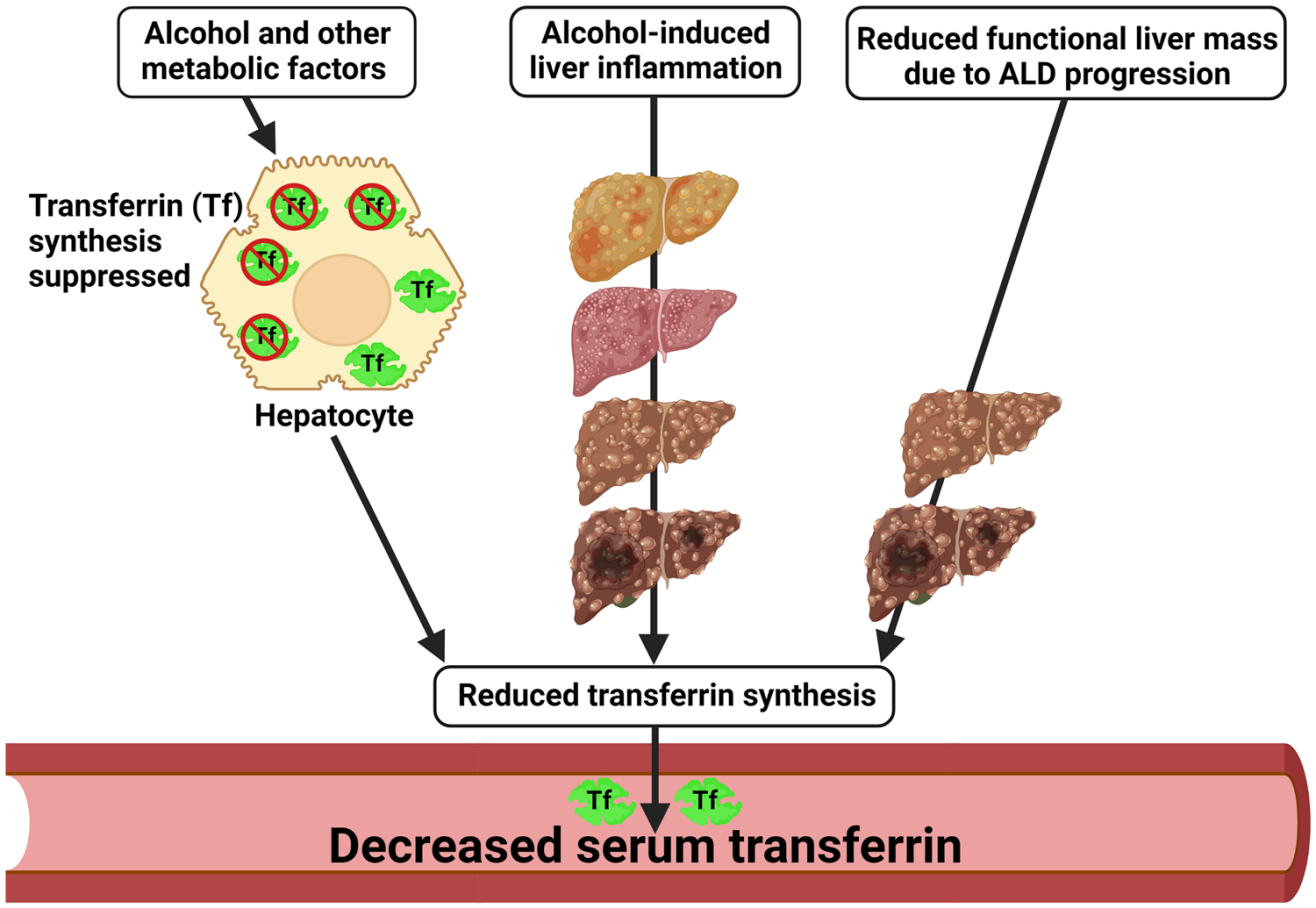
Putative reasons for alcohol-induced decrement in serum transferrin. During early ALD when there is no major liver dysfunction, alcohol and other metabolic factors such as malnutrition and vitamin A deficiency may suppress hepatic (hepatocyte) transferrin synthesis [55, 91, 92] and thereby reduce transferrin in circulation. As ALD progresses, inflammation may also decrease transferrin synthesis by hepatocytes [93]. In the later stages of ALD when there is significant liver damage, reduction in liver mass and/or the number of functional hepatocytes may be the main reason for decreased serum transferrin levels. The figure was created with BioRender.com

Low transferrin concentration in ALD suggests that alcohol and metabolic factors can directly suppress transferrin synthesis in the hepatocytes, the principal source of circulating transferrin. However, the mechanism by which this occurs is unknown. It is possible that in the early stages of ALD when there is minimal hepatic damage, alcohol-induced suppression of transferrin synthesis is the sole reason for reduced serum transferrin. However, as ALD progresses and liver scarring occurs, low transferrin levels could be due to increased inflammation (inflammation reduces transferrin levels) and/or cirrhosis and reduction in the functional liver mass caused by alcohol-induced liver damage [55] (Fig. 3).

## Alcohol, iron deficiency, and risk of infection

While high levels of alcohol consumption can cause anemia, it has been proposed that consumption of up to 2 alcoholic drinks per day can reduce the risks of iron deficiency and iron deficiency anemia by 40%. This was supported by the observation that there were very few persons with iron deficiency anemia in those with heavy alcohol consumption [12]. Wine has high iron levels and can increase iron absorption [10].

Generally, excess iron can increase the risk of infection/promote infection, and therefore, the body resorts to iron scavenging mechanisms during the early stages of infection or inflammation to deprive the microorganisms of iron and thereby control the spread of infection. Iron levels return to normal within 7–10 days [56]. However, a study involving patients with alcoholic hepatitis showed that iron was independently associated with infection within 90 days, and for every 1 µmol/L increase in iron, the risk of infection reduced by 4.2%. Thus, it was suggested that iron deficiency in ALD patients can be used to predict the risk of infection [21]. The reported reduction in the risk of infection with increment in iron reflects the requirement of iron in enhancing immunity.

The concept of reduction in the risk of infection with increment in iron is indeed the opposite to aforementioned concepts of iron-mediated facilitation of infection and infection control via iron-scavenging mechanisms. This contradiction is not surprising because both low and high iron levels can increase the risk of infection [57], reiterating the significance of iron homeostasis in the body.

## Effect of alcohol on other iron parameters

Increased serum iron can often be reflected as increased serum ferritin and/or increased iron-saturation of transferrin.

### Ferritin

Ferritin is the iron storage protein that is present both intracellularly and in the circulation. It increases in response to iron elevation and inflammation. In ALD, ferritin levels may increase and hyperferritinemia may ensue (Table 1) (which can be reversed in about 15 days) [58, 59].

**Fig. 4:**
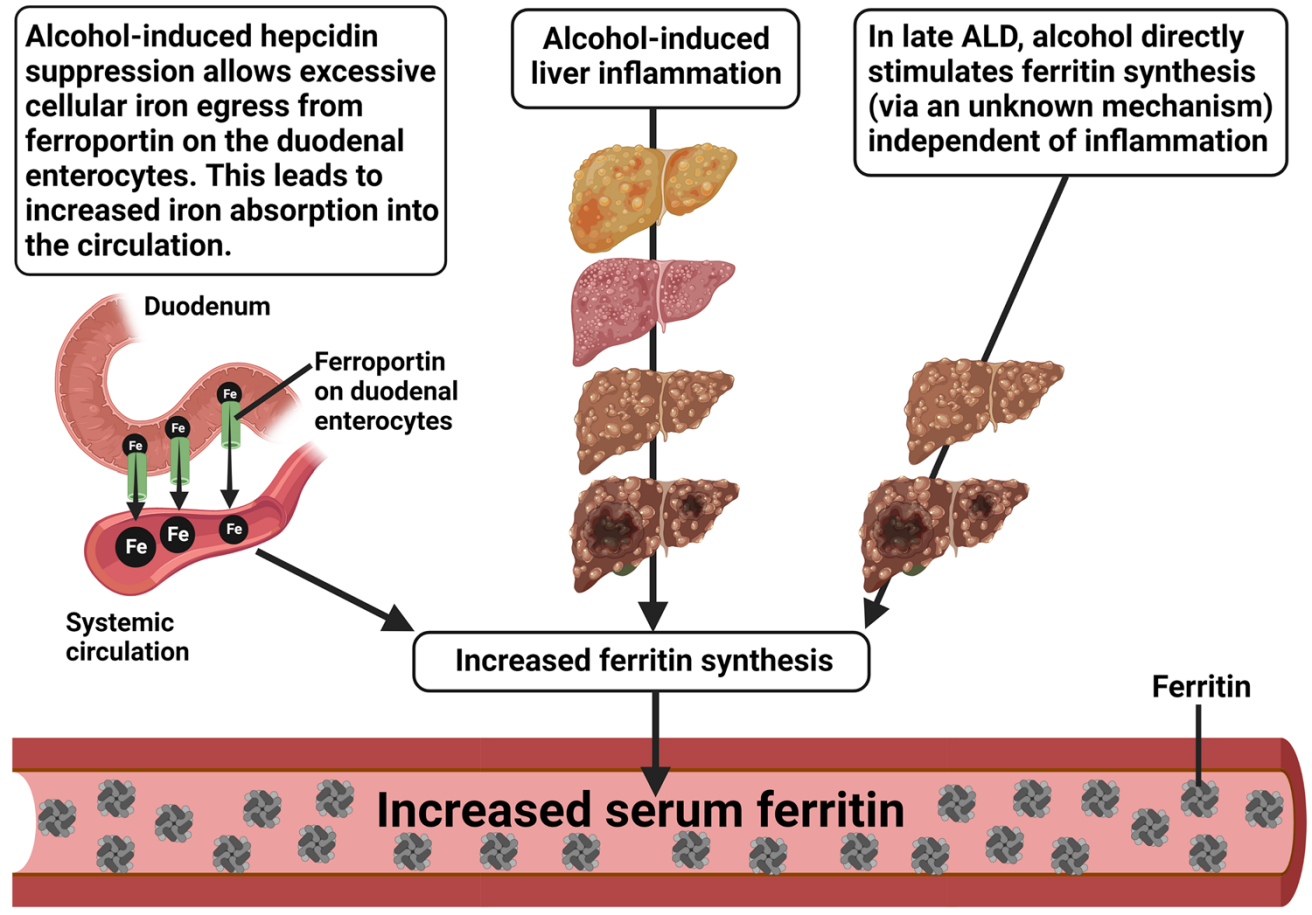
Putative reasons for alcohol-induced increment in serum ferritin. During the early stages of ALD, increased systemic iron levels followed by increased cellular iron content increase ferritin synthesis. Upon disease progression, inflammation may additionally trigger ferritin synthesis. In the later stages of ALD, alcohol may directly stimulate (de novo) ferritin synthesis via an unknown mechanism. The figure was created with BioRender.com

The alcohol-induced reduction of hepcidin, which increases serum iron levels may partly contribute to ferritin elevation in the serum (Table 1) and may be the main cause of increment in hepatic iron stores observed in approximately 50% of ALD patients [60]. Thus, elevation in serum ferritin could be a response to increment in serum iron levels, but it could also be due to the direct alcohol-induced stimulation of de novo ferritin synthesis via an unknown mechanism and/or due to alcohol-induced inflammation, as often seen in ALD [58, 61] (Fig. 4). Events like acute liver injury, inflammation, infection, and malignant disease can also elevate serum ferritin [62, 63].

In severe alcoholics, ferritin was related to inflammatory cytokines IL6 and IL8 but not to liver function impairment [53]. Thus, in healthy people and alcoholics with minimal liver disease, serum ferritin concentrations reflect total body iron stores (as measured by liver iron concentration) [64]. Here, low serum ferritin concentrations reflect iron depletion, and ferritin is used for diagnosing iron deficiency [65]. However, in alcoholics with a significant liver disease, serum ferritin levels do not reflect total body iron stores [63]. This is primarily because increased serum ferritin in alcoholic liver damage reflects hepatic inflammation and necrosis, thereby showing no association of ferritin with liver iron stores in this situation [64]. In some alcohol consumers, serum ferritin decreased following abstinence for 1.5 to 6 weeks, reiterating the effect of alcohol on ferritin [46].

### TSAT

Transferrin saturation (TSAT) reflects how much iron is bound to transferrin, i.e., how much iron is bioavailable to the cells. TSAT of less than 20% suggests iron deficiency while TSAT of more than 50% suggests iron overload [66].

Some ALD patients showed TSAT levels (and serum iron levels) similar to that in controls (Table 1). However, TSAT increased (Table 1) in patients with severe alcoholic hepatitis [54], in male adolescents following alcohol consumption [10] and in individuals who consumed more than two alcoholic drinks per day [12]. A male patient with ALD diagnosed with ferroportin disease was found to have a TSAT of 90.5% [67]. Collectively, this highlights the wide range of TSAT levels observed among ALD patients.

### CDT

Human transferrin is a glycoprotein produced by the liver hepatocytes [66]. Continuous and heavy alcohol consumption inhibits normal glycosylation of transferrin leading to the generation of carbohydrate-deficient transferrin (CDT).

CDT is elevated due to chronic alcohol consumption (Table 1) [20, 68]. It is expressed as a percentage and considered as the most specific serum biomarker for heavy alcohol consumption (defined as more than or equal to 350–420 g alcohol per week or more than 60 g per day) [69]. A CDT value of ≥ 1.7% units suggests harmful alcohol intake [70]. CDT is not significantly affected by medication, except in immunosuppressed individuals who may show decreased CDT levels. It is considered superior to the regular markers of alcohol abuse like gamma-glutamyltransferase (GGT) and mean corpuscular volume (MCV) and finds its application in cases of relapses after withdrawal, reapplying for license after driving following alcohol intake, separating patients with enzyme-induced medication from alcohol consumers and congenital disorders of glycosylation.

However, CDT usage shows limitations. Firstly, compared to males, it is less elevated in females after alcohol consumption. Also, while it can differentiate the heavy drinkers from non-drinkers and social drinkers, it cannot separate social drinkers from non-drinkers. Moreover, it cannot detect binge drinking. Iron deficiency can increase serum CDT concentration, and this can lead to false positives [59]. Most importantly, it shows relatively poor sensitivity (44–94%) and false positives in patients with severe liver conditions in the absence of alcohol use. Thus, it is not suitable for general screening for alcohol abuse. However, CDT in combination with GGT and MCV holds great promise for detecting chronic alcohol consumption [2, 69–73].

### sTfR

Circulating iron-bound transferrin supplies iron to various cells by interacting with transferrin receptor-1 (TfR1) on cell-surfaces. Iron-bound transferrin binds to TfR1 and forms the transferrin-TfR1 complex. The complex is internalized into an endosome, iron from the complex is released into the cytosol, and the resulting iron-devoid transferrin-TfR1 complex returns to the cell-surface for more iron uptake. Transferrin returns to the circulation, and TfR1 is recycled [66].

By convention, soluble transferrin receptor (sTfR) refers to the cleaved extracellular region of the membrane-bound TfR1. sTfR is found in the circulation, and its concentration reflects the expression of membrane-bound TfR1 [74]. sTfR in the serum is predominantly derived from the developing RBCs because in healthy individuals, over 80% of cellular transferrin receptor mass is present in the bone marrow [20]. Therefore, sTfR is related to the degree of erythropoiesis and cellular iron demand. In iron deficiency, developing RBCs increase their TfR1 expression (aiming to obtain more iron) resulting in higher concentration of sTfR in the circulation. Thus, sTfR is used as a biomarker of iron deficiency, and it is used to distinguish between iron deficiency anemia and anemia of inflammation [74].

Studies have shown that there was not much difference in sTfR concentrations in health and ALD (Table 1). There could be several reasons for this. First, here, the patients did not show iron deficiency [20]. This did not necessitate TfR1 upregulation on cell membranes, thereby minimizing the possibility of sTfR shedding in the circulation. Indeed, ALD patients have shown increased hepatic TfR1 expression [75], but this would be to facilitate alcohol-induced liver iron uptake and would not lead to shedding of sTfR in the circulation. The second reason may be the insensitivity of sTfR to inflammation. ALD is characterized by inflammation, but unlike ferritin, sTfR is not an acute phase protein and is not responsive to inflammation, i.e., its levels remain unchanged during inflammation. This feature of sTfR is used to distinguish between iron deficiency anemia and the anemia of inflammation/chronic disease [76]. Thus, sTfR levels may not necessarily differ between healthy and ALD subjects.

However, apparently, sTfR was found to be higher in some frequent drinkers (alcohol dependents) than social drinkers (non-dependents on alcohol) [68]. This increment could reflect the onset of iron deficiency anemia in these subjects.

## The differing pictures of iron parameters among alcohol consumers

### The “iron norm”, and alcohol consumers following and defying the norm

Under physiological conditions, decrement in serum hepcidin leads to increased intestinal iron absorption and iron egress into the circulation from the iron storing cells [28]. Accordingly, excess-iron–induced increases in serum ferritin and TSAT are expected at some point in time.

Several alcohol-related studies have demonstrated the aforementioned interrelationships between iron parameters and conformed to the iron norm, either fully or partly. For example, increase in body iron stores in chronic alcohol consumers/alcohol dependents were reflected as increases in ferritin (approximately 69% of subjects) [59, 77] and TSAT (> 62% in approximately 15% of subjects) [77]. However, several subjects in these studies did not show ferritin and TSAT elevation. Also, in another study, ALD patients showed low serum hepcidin, but there were no significant alterations in the levels of serum iron, serum ferritin, and TSAT [22], defying the expected effect of low hepcidin on serum iron levels. Collectively, this suggests that all alcohol consumers do not follow the iron norm and do not show the same effect on iron parameters (Table 1).

### Ferritin in context

Studies have shown alcohol consumers with low hepcidin mRNA expression but unaltered serum iron levels along with increased serum ferritin and TSAT [25]; similar levels of serum iron to controls, but higher serum ferritin and TSAT [20]; and lower levels of total iron but higher levels of serum ferritin and similar levels of TSAT index, compared to controls [53]. Elevation of serum ferritin in the absence of serum iron loading, as seen in these studies, may be due to inflammation or alcohol-induced de novo ferritin synthesis, as discussed previously.

### Possible reasons for the observed differences in iron parameters between alcohol consumers

Essentially, level of the same iron parameter could differ between alcohol consumers (Table 1). This could be due to differences in their health status (including intestinal health) and the disease stage at which these iron parameters were measured. It is noteworthy that serum iron levels can vary every hour and can vary between individuals. Generally, it is not greatly reliable as a sole marker because it fluctuates and alters due to infection and intake of iron prior to the time of measurement [78]. This could be an additional reason for the observed differences.

Amount and frequency of alcohol consumption and gender (proportion of males and females in the study) may contribute to the observed differences in iron parameters in response to alcohol (Table 1). For example, in the study by Scotet et al. hemochromatosis (C282Y mutation) patients who consumed more than or equal to 60 g alcohol/day showed higher levels of serum iron, serum ferritin, and TSAT than those who consumed less alcohol. In this study, majority (85.2%) of hemochromatosis patients drank alcohol, either occasionally or frequently. Males showed significantly higher levels of serum iron, ferritin, and TSAT compared to females, showcasing gender-based differences [79]. Similarly, in another study, male adolescents who drank frequently showed significantly higher serum iron and TSAT than non-drinking male adolescents, but the latter was not the case with female adolescent drinkers [80].

## Alcohol consumption and hemochromatosis

Hemochromatosis is a disease of genetic origin. It is caused by deficiency or impairment in hepcidin synthesis or function resulting from mutations in the genes that regulate hepcidin synthesis or function. The mutations lead to increased intestinal iron absorption and release of iron from the iron-storing macrophages resulting in elevated systemic iron levels that are followed by iron deposition in various organs. Thus, hemochromatosis is characterized by elevated TSAT and progressive iron loading in several organs (mainly the liver) in the absence of anemia and/or reticulocytosis [81].

While hemochromatosis can occur due to mutations in the non-*HFE* genes, a common cause is the inheritance of C282Y mutation in the *HFE* gene. Excessive alcohol consumption in C282Y homozygous patients elevates iron parameters such as serum ferritin, serum iron, and transferrin saturation (Table 1). In such patients, excess alcohol has an additive hepatotoxic effect that can aggravate liver fibrogenesis and increase the risk of developing cirrhosis and hepatocellular carcinoma [79]. Thus, excessive alcohol consumption by these patients can worsen disease severity. No wonder, hemochromatosis patients that consumed more than 60 g/day of alcohol were almost 9 times more likely to develop liver cirrhosis than those who drank less [82]. This explains the higher prevalence of liver fibrosis and cirrhosis in patients diagnosed with both hemochromatosis and alcoholism, compared to non-alcoholic patients with hemochromatosis [83].

## Summary

**Fig. 5:**
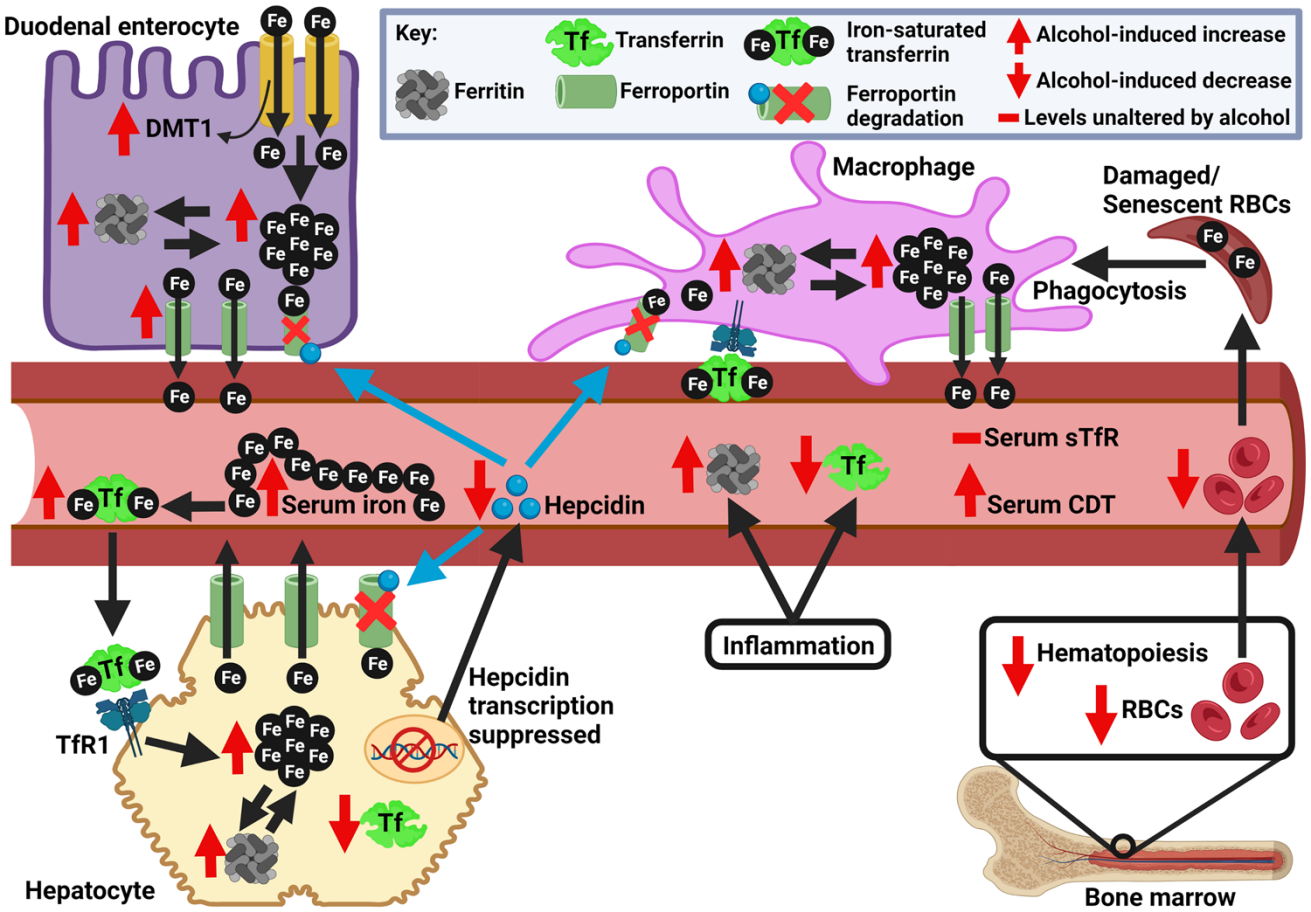
Overview of alcohol-induced alterations in iron parameters. The figure shows alcohol-induced alterations in iron parameters, as indicated via red upward and downward arrows. Essentially, alcohol decreases hepcidin synthesis, which causes decrement in serum hepcidin. This allows excessive iron absorption into the circulation via the duodenal enterocytes and increased iron egress from the iron storing macrophages and hepatocytes leading to elevation in serum iron levels. Serum iron is also partly elevated due to alcohol-induced upregulations of duodenal DMT-1 and ferroportin that facilitate iron entry and exit into and from the enterocyte, respectively. Resultantly, intracellular and serum ferritin levels increase and so does the iron saturation of serum transferrin. Interestingly, ferritin is elevated by both increased iron levels and inflammation, the latter often found in heavy or chronic alcohol consumers. Inflammation also reduces transferrin synthesis. Alongside, alcohol can decrease hematopoietic activity in the bone marrow leading to reduction in the number of functional red blood cells (RBCs), thereby contributing to the many causes of anemia, as observed in some ALD cases. Carbohydrate-deficient transferrin (CDT) levels increase, but alcohol does not seem to alter soluble transferrin receptor (sTfR) levels. Distinct from the depiction in this figure, serum iron levels may decrease or remain unaltered in ALD. Also, levels of transferrin and transferrin saturation may remain unaltered. Hemoglobin levels (not shown in the figure) may increase, decrease, or remain unaltered. The figure was created with BioRender.com

The effect of alcohol on iron and iron-related proteins has been summarized in Fig. 5. Essentially, alcohol may increase or decrease the levels of hemoglobin and serum iron or show no effect on these iron parameters. Alcohol-induced increments in intestinal iron absorption, serum ferritin, and carbohydrate-deficient transferrin have been reported. Alcohol tends to decrease the levels of serum hepcidin and transferrin; the latter may remain unchanged, while transferrin saturation may increase or remain unaltered. Soluble transferrin receptor has been reported to remain unaltered. Notably, some ALD cases may show anemia, and this could be due to erythropoiesis disruption or gastrointestinal bleeding in cases of alcohol-induced cirrhosis or reduced serum transferrin.
